# Lectin–Rose Bengal Conjugates for Targeted Photodynamic Inactivation of Pathogenic Bacteria

**DOI:** 10.3390/ijms27020819

**Published:** 2026-01-14

**Authors:** Melad Atrash, Iryna Hovor, Marina Nisnevitch, Faina Nakonechny

**Affiliations:** Department of Chemical Engineering, Ariel University, Kiryat-ha-Mada, Ariel 4070000, Israel

**Keywords:** photosensitizers, Rose Bengal, lectins, conjugation, *Pisum sativum* agglutinin, *Laburnum anagyroides* agglutinin, photodynamic antibacterial chemotherapy

## Abstract

The growing threat of antibiotic-resistant bacteria necessitates the development of alternative antimicrobial strategies. This study investigated the design and evaluation of novel photodynamic agents based on Rose Bengal (RB) conjugated to two plant lectins, *Pisum sativum* agglutinin (PSA) and *Laburnum anagyroides* agglutinin (LABA), for targeted photodynamic inactivation of Gram-positive and Gram-negative bacteria. Both conjugates demonstrated high singlet oxygen quantum yields compared with free RB. Antibacterial efficacy was assessed against methicillin-sensitive and methicillin-resistant *Staphylococcus aureus*, *Escherichia coli*, *Pseudomonas aeruginosa*, and *Salmonella paratyphi* B under white LED illumination. PSA-RB exhibited superior bactericidal activity against all strains, whereas LABA-RB showed strain-specific efficacy, particularly against Gram-negative species. A binary mixture of PSA-RB and LABA-RB synergistically inactivated both MSSA and MRSA at RB concentrations of 6–10 nM and light doses of 3.1–7.8 J/cm^2^. Complete killing of *E. coli* and *S. paratyphi* B was achieved at approximately half the RB concentrations needed for individual conjugates. PSA-RB activity primarily drove the inactivation of *P. aeruginosa*. Uptake studies revealed significantly enhanced accumulation of lectin-conjugated RB compared to free RB, with synergistic uptake observed for the conjugate mixture. These results suggest that lectin-based RB conjugates are effective antibacterial agents for photodynamic treatment, especially via the dual-targeting method.

## 1. Introduction

Antibiotic resistance in bacteria is a significant problem in modern medicine, driven by the ability of microorganisms to develop defense mechanisms against antibiotics [1]. The development of resistance compromises the effectiveness of many antibiotics, necessitating the development of alternative or complementary therapeutic approaches [2]. One method for combating antibiotic resistance is photodynamic antibacterial chemotherapy (PACT), which relies on the specific accumulation of photosensitizers in pathogenic cells, followed by light activation and the formation of cytotoxic reactive oxygen species (ROS) [3,4]. This method is highly selective and has minimal systemic side effects. However, the effectiveness of PACT largely depends on the ability of the photosensitizer to penetrate and accumulate in target cells [5,6]. In this context, the use of lectins as biologically active molecules for the targeted delivery of photosensitizers represents an innovative solution. Lectins are carbohydrate-binding proteins that can selectively recognize specific carbohydrate structures on the cell surface [7,8]. This selectivity ensures targeted binding of the photosensitizer to pathogenic microbial cells, increasing the effectiveness of PACT. Unlike free photosensitizers, lectin–photosensitizer conjugates exhibit greater efficacy due to improved target accumulation, which provides a more potent and targeted photodynamic effect [9,10]. Additionally, lectins help overcome structural barriers in microorganisms, such as the lipopolysaccharide layer of Gram-negative bacteria, which hinders the access of many antimicrobial agents [11]. Thus, the use of lectin–photosensitizer conjugates significantly expands the capabilities of PACT, improving its clinical potential for the treatment of antibiotic-resistant infections.

This study explored the use of Rose Bengal (RB) conjugated with two plant lectins for photodynamic antibacterial chemotherapy against pathogenic bacteria. Rose Bengal is a xanthene-based photosensitizer that has been extensively studied in photodynamic therapy (PDT) because of its high quantum yield of singlet oxygen generation and strong absorption in the visible region [12]. Upon light activation, RB efficiently produces reactive oxygen species (ROS), primarily singlet oxygen, which can induce oxidative damage to cellular membranes, proteins, and nucleic acids, leading to rapid microbial inactivation [13,14]. Owing to these properties, RB has been widely applied in antimicrobial photodynamic inactivation against bacteria, fungi, and biofilms, including antibiotic-resistant strains [13,14,15,16]. However, the clinical and practical use of free RB is limited by its hydrophilic nature, poor selectivity toward microbial cells over host tissues, and restricted penetration across bacterial envelopes—particularly in Gram-negative bacteria with complex outer membranes [17,18]. These limitations have motivated the development of targeted RB delivery systems, including conjugation to biomolecules or carriers, to increase local accumulation at microbial surfaces and improve photodynamic efficacy while reducing the required photosensitizer concentration and light dose [9,10,19,20,21]. The lectins were selected for their complementary carbohydrate specificities. Lectin PSA (*Pisum sativum* agglutinin) exhibits binding specificity for both glucose and α-mannose because its active site recognizes the similar spatial arrangement of the hydroxyl groups in these C2-epimers [22,23]. The LABA (*Laburnum anagyroides* agglutinin) lectin has an active site that specifically interacts with the unique structural elements of L-fucose, primarily recognizing and exploiting the absence of the hydroxyl group at the C6 position of the sugar ring [24,25].

The present study specifically aims to compare the photodynamic efficacy of lectin-conjugated RB photosensitizers with that of free RB in the targeted eradication of Gram-positive and Gram-negative bacteria. Several bacterial strains, including *S. aureus*, MRSA (Gram-positive), *E. coli*, *P. aeruginosa*, and *S. paratyphi* B (Gram-negative), were chosen to represent a range of clinically significant human pathogens and to investigate the interactions of selected lectins with bacterial cell walls of various compositions and structures. The inclusion of MRSA and the major Gram-negative species *P. aeruginosa* and *E. coli* enables a thorough evaluation of the ability of lectin conjugates to penetrate and kill microorganisms resistant to traditional treatments, as well as bacteria protected by a robust LPS layer. Lectins with varying carbohydrate-binding affinities were chosen to evaluate the efficacy of PACT via individual lectin–RB conjugates and to test the synergistic effect of the PSA-RB/LABA-RB binary system. We hypothesized that this mixture would allow simultaneous, non-competitive binding to different bacterial surface glycans, increasing the total localized RB concentration compared with that of either conjugate alone. This complementary binding could also distribute RB more uniformly across the cell surface, maximizing ROS exposure and synergistically enhancing PACT efficacy.

## 2. Results and Discussion

### 2.1. Microscopic Analysis of PSA and LABA Binding to Gram-Positive and Gram-Negative Bacteria

**Figure 1:**
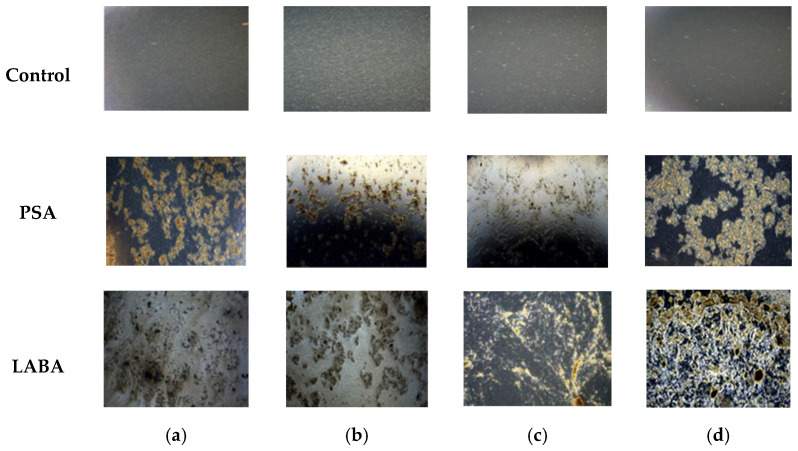
Visualization of interaction between PSA (second line) or LABA (third line) and Gram-positive and Gram-negative bacteria: (**a**) *S. aureus*, (**b**) *E. coli*, (**c**) *P. aeruginosa*, and (**d**) *S. paratyphi B*. The bacterial cells were incubated with PSA or LABA for 2 min and immediately examined using an optical microscope at ×400 magnification. The first line presents control cells.

To study the binding specificity of the lectins PSA and LABA to bacterial cell walls, agglutination assays were performed for a short period and examined under an optical microscope using Gram-positive and Gram-negative bacteria. PSA causes pronounced agglutination of Gram-positive (*S. aureus*, Figure 1a) and Gram-negative (*E. coli*, *P. aeruginosa*, and *S. paratyphi* B, Figure 1b–d) bacterial cells, whereas control cells remain intact. LABA also agglutinated all the tested bacteria, with the strongest effect observed in the case of *S. paratyphi* B (Figure 1d). These observations indicate that both lectins exhibit good affinity for all the tested bacteria, and we assume that both lectins can be used as bacterial targeting agents in photodynamic applications, which is consistent with the targeted strategy demonstrated in our previous study using RB–lectin conjugates [9].

### 2.2. Synthesis and Characterization of Lectin–RB Conjugates

**Scheme 1:**
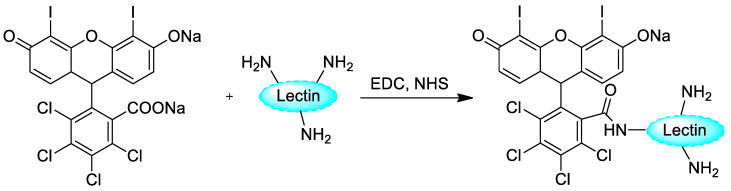
Schematic representation of the EDC/NHS-mediated conjugation of Rose Bengal to lectins.

The conjugates PSA-RB and LABA-RB were prepared by covalent bonding of Rose Bengal (RB) to the amino groups of lectins through EDC/NHS-mediated amine coupling, using a previously described method [9]. The conjugation strategy and the formation of covalent amide bonds between RB and lectin amino groups are illustrated in Scheme 1.

**Figure 2:**
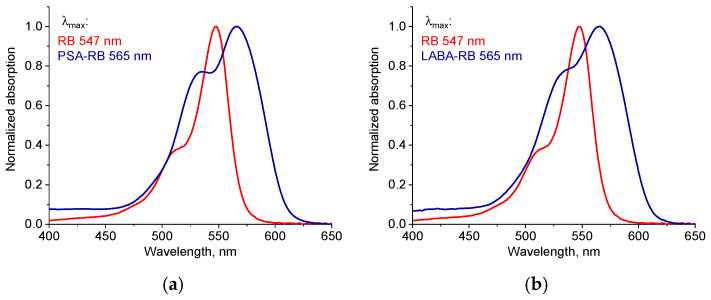
Absorption spectra of lectin–RB conjugates: PSA-RB (**a**) and LABA-RB (**b**) in 0.1 M PBS, pH 7.4.

Successful conjugation was validated by characteristic alterations in the UV spectra (Figure 2). A red shift of 18 nm in the absorption maxima of both conjugates, along with a noticeable broadening of the absorption spectra and a decrease in the monomer-to-aggregate absorption ratio of RB from 2.63 for free RB (A547/A514) to only 1.29 (A565/A537) with lectins, was observed (Figure 2).

**Figure 3:**
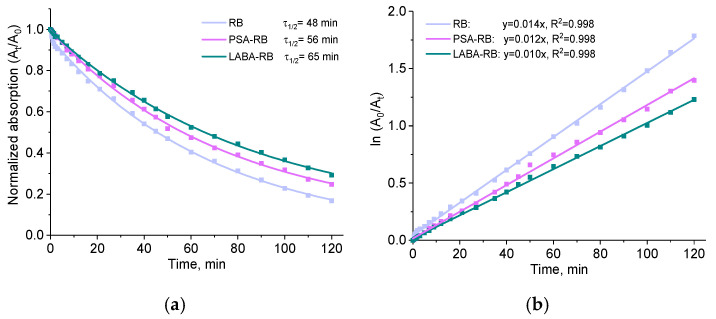
Photostability (**a**) and time-dependent photodecomposition (**b**) of RB, PSA-RB, and LABA-RB in 0.1 M PBS, pH 7.4, under light irradiation by an LED lamp.

The singlet oxygen quantum yields (Φ_Δ_) of the resulting conjugates were measured to evaluate their photodynamic activity (Figure 3). These yields were calculated using 9,10-anthracenediyl-bis(methylene)dimalonic acid (ABMDMA) as a scavenger of the reactive oxygen species generated. Free RB has a high value of Φ_Δ_ = 0.76, but upon conjugation, Φ_Δ_ decreased slightly: the quantum yield was 0.72 for PSA-RB and 0.70 for LABA-RB. These data indicate that the conjugates still possess substantial potential for singlet oxygen formation upon labeling.

The photostability of the samples was evaluated under continuous visible-light irradiation by monitoring the temporal decrease in their absorption maxima (Figure 3).

Free RB exhibited a photostability lifetime of 48 min, whereas the conjugates PSA-RB and LABA-RB presented values of 56 min and 65 min, respectively (Figure 3a). Compared with the free dye, the conjugation of RB to the lectins PSA and LABA results in increased photostability. The kinetic constants of photodecomposition (*k*), obtained from the slopes of the ln(A_0_/A_t_) vs. time plots, were 0.014 min^−1^ for RB, 0.012 min^−1^ for PSA-RB, and 0.010 min^−1^ for LABA-RB (Figure 3b).

The enhanced photostability observed in the RB–lectin conjugates is primarily driven by the protein’s dual protective mechanism. Its bulky structure provides steric shielding, separating the chromophore from destructive oxidative radicals while simultaneously limiting molecular mobility. This constraint minimizes non-radiative decay pathways responsible for photobleaching, allowing the conjugates to maintain high photodynamic efficiency. The photostability data suggest that these RB–lectin conjugates could be efficient and stable photosensitizers under prolonged exposure to light.

### 2.3. The Photodynamic Activity of the RB–Lectin Conjugates

We evaluated the antibacterial photodynamic properties of PSA-RB, LABA-RB, and free RB against Gram-positive and Gram-negative bacteria. After the conjugates and free RB were added to the bacterial cell cultures, the samples were incubated either in the dark or exposed to white LED light. In parallel series, the concentrations of RB in the lectin–RB conjugates and of the free RB were equal.

#### 2.3.1. Antibacterial Activity Against Gram-Positive Bacteria

**Figure 4:**
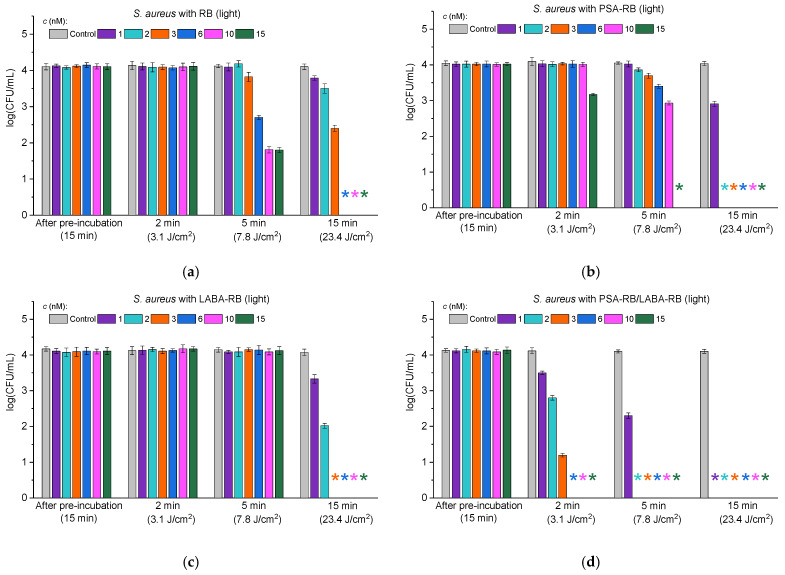
Effects of free RB (**a**), PSA-RB (**b**), LABA-RB (**c**), and a mixture of PSA-RB and LABA-RB (1:1) (**d**) on *S. aureus* cells illuminated by white LED. Error bars represent the STDEV. The asterisk * indicates the total inhibition of bacterial cells.

In the first step, we tested the effect of free RB on Gram-positive *S. aureus* (MSSA) in the dark and under illumination by visible light (Figure 4). Free RB had no dark toxic effect at any of the tested concentrations, ranging from 1 to 15 nM. However, when exposed to light, the cells were completely inhibited by RB at a concentration of 6 nM after 15 min of illumination (Figure 4a).

As in the case of free RB, neither PSA-RB, LABA-RB, nor their mixture exhibited any dark toxicity. Under illumination, free RB eradicated *S. aureus* cells at concentrations of 6 nM and higher after 15 min. PSA-RB completely inactivated *S. aureus* cells at a concentration of 15 nM after 5 min (Figure 4b), whereas LABA-RB was not effective under these conditions, and free RB showed a reduction in the concentration of approximately 1.8 log_10_. The antibacterial activity of LABA-RB became apparent only after 15 min of irradiation, when complete inactivation of *S. aureus* was observed at a concentration of 3 nM (Figure 4c). The latter conjugate is less effective than PS-RB but more effective than RB, which completely kills bacteria at a concentration of 6 nM. Each of the conjugates was more active than free RB, although the difference was not statistically significant, with *p* values of 0.0997 for PSA-RB and 0.243 for LABA-RB. These observations suggest that the α-mannose/glucose structures recognized by PSA represent a more critical or accessible target on the *S. aureus* cell wall than the L-fucose structures targeted by LABA.

The PSA-RB/LABA-RB mixture demonstrated a powerful synergistic system (Figure 4d), causing complete microbial inactivation at an exceptionally low concentration of 6 nM after just 2 min of irradiation (3.1 J/cm^2^). This remarkable finding contrasts sharply with the performance of the individual agents, as neither free RB nor the individual conjugates showed any measurable bactericidal efficacy at the same low initial radiation dose and the same concentration of photosensitizer. The difference between the activities of the conjugate mixture and free RB was considerable (*p* value of 0.00159).

#### 2.3.2. Antibacterial Activity Against Multi-Drug-Resistant Bacteria

**Figure 5:**
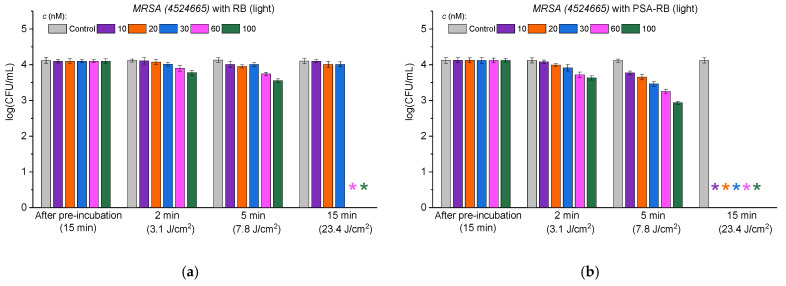

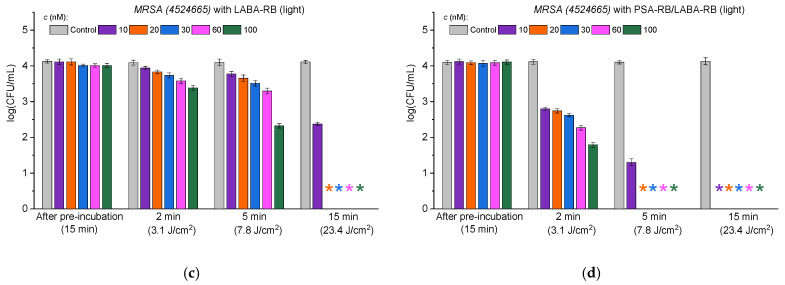
Effects of free RB (**a**), PSA-RB (**b**), LABA-RB (**c**), and the mixture of PSA-RB/LABA-RB (1:1) (**d**) on MRSA 4524665 cells illuminated by white LED. Error bars represent the STDEV. An asterisk * indicates the total inhibition of bacterial cells.

**Figure 6:**
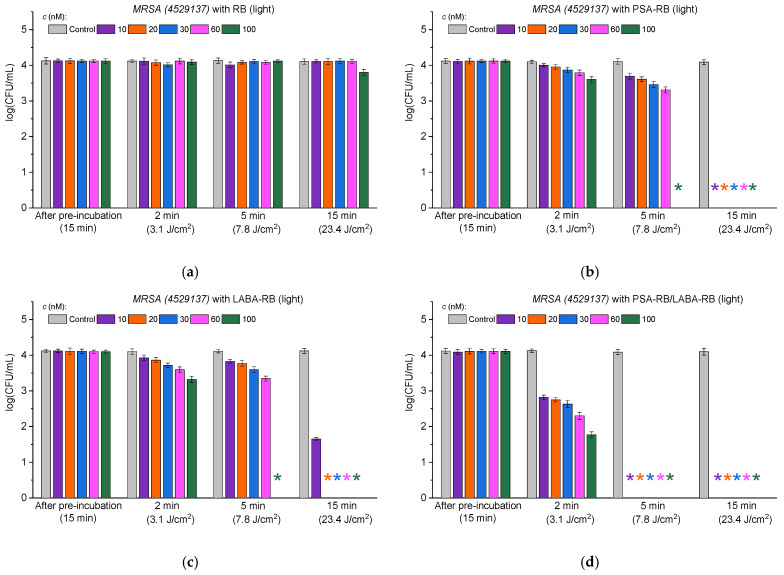
Effects of free RB (**a**), PSA-RB (**b**), LABA-RB (**c**), and the mixture of PSA-RB/LABA-RB (1:1) (**d**) on MRSA 4529137 cells illuminated by white LED. Error bars represent the STDEV. An asterisk * indicates total inhibition of bacterial cells.

To determine whether the RB–lectin conjugates are effective against drug-resistant bacteria, we tested them on two clinical MRSA isolates: 4524665 and 4529137 (Figure 5 and Figure 6). According to clinical reports, MRSA 4524665 is resistant to methicillin, oxacillin, and penicillin, whereas MRSA 4529137 shows broader resistance, including erythromycin, clindamycin, gentamicin, and ofloxacin, with intermediate sensitivity to ceftaroline. We examined how each strain responded to treatment with free RB (panels “a” in Figure 5 and Figure 6), PSA-RB (panels “b” in Figure 5 and Figure 6), LABA-RB (panels “c” in Figure 5 and Figure 6), and the mixture of PSA-RB/LABA-RB (panels “d” in Figure 5 and Figure 6) in the dark and under illumination at different concentrations.

The free RB activity was low when MRSA 4524665 was killed by RB at a concentration of 60 nM after 15 min of illumination, whereas the strain MRSA 4520137 was not completely inactivated, even under the maximal dose of 100 nM at 23.4 J/cm^2^. However, the PSA-RB conjugate generally proved to be a more potent agent, requiring a lower concentration than LABA-RB for both strains. In both resistant strains, PSA-RB was significantly more active than free RB (the *p* value for MRSA 4524665 was 0.0399, and that for MRSA 4520137 was 3.59 × 10^−9^), and LABA-RB also had higher activity (the *p* value for MRSA 4524665 was 0.0826, and that for MRSA 4520137 was 1.09 × 10^−4^). These findings are consistent with previous reports, which show that, compared with free dyes, lectin–photosensitizer conjugates can significantly enhance photodynamic inactivation because of improved bacterial binding and more efficient delivery of the photosensitizer to the cell surface [9].

The PSA-RB/LABA-RB mixture, as in the case of MSSA, exhibited the highest efficacy against both MRSA strains, demonstrating a strong synergistic effect. This mixture completely inactivated bacteria under a light dose of 7.8 J/cm^2^ at 20 nM and 10 nM for MRSA 4524665 and MRSA 4529137, respectively. The *p* values calculated for the difference between free RB and the conjugate mixture were 1.8 × 10^−8^ and 3.59 × 10^−12^ for MRSA 4524665 and MRSA 4529137, respectively. These findings confirm that the complementary targeting of multiple cell wall glycans is a good strategy for minimizing the required light dose against MRSA.

#### 2.3.3. Antibacterial Activity Against Gram-Negative Bacteria

Next, the efficacy of PSA-RB and LABA-RB was assessed against three Gram-negative species: *Escherichia coli*, *Pseudomonas aeruginosa*, and *Salmonella paratyphi* B. This part of our study was particularly important, as it is well known that Gram-negative bacteria are less responsive to photodynamic treatment than Gram-positive bacteria [26,27].

**Figure 7:**
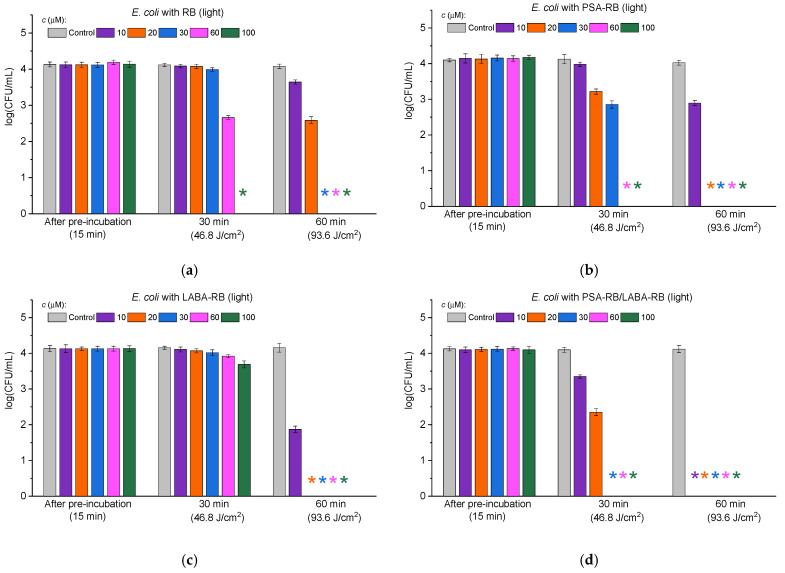
Effects of free RB (**a**), PSA-RB (**b**), LABA-RB (**c**), and a mixture of PSA-RB and LABA-RB (1:1) (**d**) on *E. coli* cells illuminated by white LED. Error bars represent the STDEV. The asterisk * indicates total inhibition of bacterial cells.

**Figure 8:**
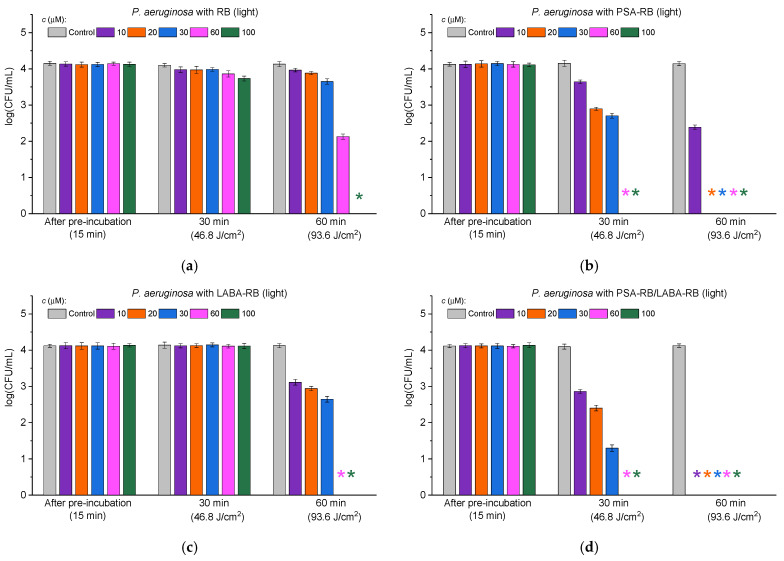
Effects of free RB (**a**), PSA-RB (**b**), LABA-RB (**c**), and a mixture of PSA-RB and LABA-RB (1:1) (**d**) on *P. aeruginosa* cells illuminated by white LED. Error bars represent the STDEV. The asterisk * indicates total inhibition of bacterial cells.

**Figure 9:**
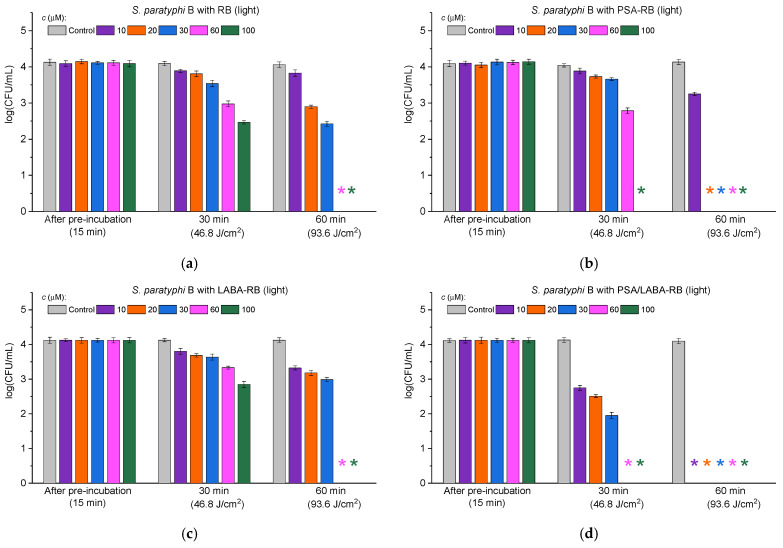
The effects of free RB (**a**), PSA-RB (**b**), LABA-RB (**c**), and a mixture of PSA-RB and LABA-RB (1:1) (**d**) on *S. paratyphi* B cells illuminated by white LED. Error bars represent the STDEV. The asterisk * indicates total inhibition of bacterial cells.

Figure 7, Figure 8 and Figure 9 show the antibacterial activity against *E. coli* (Figure 7), *P. aeruginosa* (Figure 8), and *S. paratyphi* B (Figure 9) in the case of free RB (panels “a” in Figure 7, Figure 8 and Figure 9); PSA-RB (panels “b” in Figure 7, Figure 8 and Figure 9); LABA-RB (panels “c” in Figure 7, Figure 8 and Figure 9); and the mixture of PSA-RB/LABA-RB (panels “d” in Figure 7, Figure 8 and Figure 9) at different concentrations under dark incubation and upon visible light illumination or in the dark.

Free RB exhibited very low efficacy in killing *E. coli* at a concentration of only 100 µM and a light dose of 46.8 J/cm^2^. PSA-RB is the most effective individual conjugate, achieving complete inactivation at a concentration of 60 µM at the same dose. LABA-RB showed significantly lower efficacy than PSA-RB and completely inactivated *E. coli* at only 20 µM after 60 min (93.6 J/cm^2^) of irradiation, likely because L-fucose target sites are less abundant or less critical on the *E. coli* surface. However, the PSA-RB/LABA-RB mixture demonstrated superior activity, achieving complete microbial inactivation at 30 µM for 30 min of irradiation (Figure 7). This dose reduction, compared with that of individual conjugates, strongly confirms a synergistic effect from the complementary dual targeting of the LPS layer, making it an effective PACT against *E. coli*. In contrast to Gram-positive strains, neither PSA-RB nor LABA-RB significantly increased the antibacterial activity over that of free RB, since the *p* values in comparison with those of free RB were 0.392 and 0.181, respectively. On the other hand, the conjugate mixture was more active than free RB, with a *p* value of 0.0277.

While free RB was ultimately capable of achieving complete microbial inactivation against *P. aeruginosa*, this required the highest concentration tested, 100 µM, at 93.6 J/cm^2^, confirming that its hydrophilic nature severely inhibits effective crossing of the bacterial outer membrane (Figure 8). LABA-RB was slightly more effective. For complete inactivation of bacteria, the highest dose of radiation was also needed; however, a slightly lower concentration of 60 µM was sufficient, compared to the free RB. The PSA-RB conjugate and the PSA-RB/LABA-RB mixture demonstrated equivalent maximal efficacy against *P. aeruginosa*, with complete microbial inactivation at a concentration of 60 µM after 30 min of illumination. With respect to the comparison of conjugates to free RB, statistically significant differences are observed in the cases of the PSA-RB conjugate and the PSA-RB/LABA-RB mixture, as demonstrated by *p* values of 0.00881 and 0.000217, respectively. However, the greater efficiency of LABA-RB compared to free RB has not been statistically proven (*p* value = 0.196).

This finding indicates that the high performance of the mixture is entirely dominated by the PSA-RB component, suggesting that the PSA-targeted mannose/glucose structures represent the limiting and accessible binding sites on the Gram-negative outer membrane of *P. aeruginosa*. Consequently, the addition of the less efficient LABA-RB component provides no discernible synergistic advantage, as the optimal performance threshold is already reached by the superior PSA-targeting pathway.

As with the other bacteria studied here, free RB inhibited the cells of *S. paratyphi* B at a relatively high concentration of 60 µM after prolonged illumination for 1 h (93.6 J/cm^2^) (Figure 9). The conjugation of RB with LABA did not increase its antibacterial activity against *S. paratyphi* B, and the free dye and its conjugate completely inactivated the bacteria under the same conditions. The PSA-RB conjugate demonstrated greater efficacy, inactivating bacteria at a dose of 100 µM under an illumination dose of 46.8 J/cm^2^. The PSA-RB/LABA-RB mixture exhibited a small synergistic effect, killing bacteria at a concentration of 60 µM under illumination of 46.8 J/cm^2^. For *P. aeruginosa,* the difference between the activities of free RB and PSA-RB and the conjugate mixture was statistically significant, with *p* values of 0.00786 for PSA-RB and 0.00178 for PSA-RB/LABA-RB, but there was no difference between the efficacies of free RB and LABA-RB (*p* value = 0.942).

When applied alone, PSA-RB showed the highest individual efficacy at a concentration of 60 µM and a light dose of 46.8 J/cm^2^ against *E. coli* and *P. aeruginosa*, whereas the LABA-RB conjugate was significantly less effective. The consistently highest efficacy of the PSA-RB conjugate against Gram-negative bacteria suggests that the α-mannose/glucose structures recognized by PSA play a more important role in targeting the conjugates to the cell wall or LPS surface than the L-fucose targets of LABA. This finding may also indicate that PSA increases the RB concentration near key LPS or membrane components.

The performance of the PSA-RB/LABA-RB mixture for the three tested Gram-negative species confirmed the good potential of the dual-targeting strategy; however, its synergistic effect critically depended on the availability of the target for the specific pathogen. The greatest benefit was observed against *E. coli*, where the mixture caused complete microbial inactivation at significantly lower doses (30 µM at 30 min) than required by any individual conjugate, confirming that a synergistic mechanism was crucial for low-dose PACT applications. Conversely, while achieving maximal eradication of *P. aeruginosa* (60 µM at 30 min), the performance of the mixture was entirely dominated by the PSA-RB component, suggesting that the PSA-targeted α-mannose/glucose structures represent the limiting and most accessible binding site on the *P. aeruginosa* outer membrane. Finally, the mixture of conjugates exhibited a moderate synergistic effect against *S. paratyphi* B, primarily by reducing the light dose required to inhibit bacterial growth.

These findings are consistent with previous reports showing that targeted delivery strategies are essential for improving PACT efficacy against Gram-negative bacteria [10], whose outer membrane significantly limits photosensitizer penetration [28,29].

### 2.4. Uptake of Free and Conjugated RB by Bacteria

**Figure 10:**
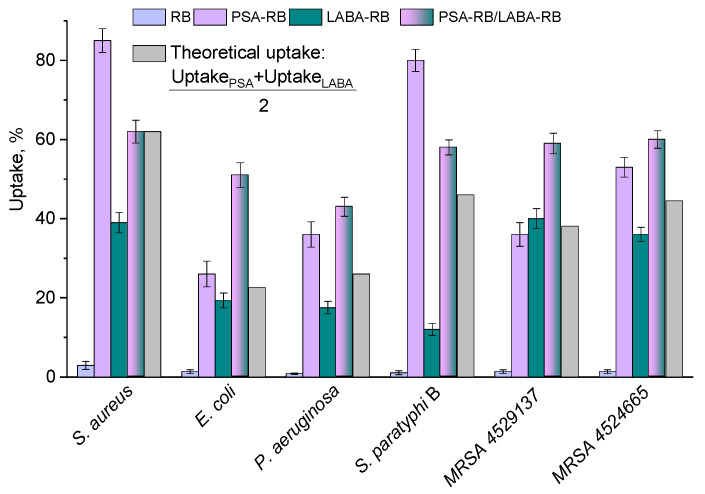
RB, PSA-RB, LABA-RB, and binary system PSA-RB/LABA-RB uptake by bacterial cells.

To evaluate the potential of the synthesized conjugates as targeted phototherapeutic agents compared with free RB, it was essential to demonstrate not only their photostability but also their cellular uptake efficiency. Enhanced and selective uptake in target cells is a prerequisite for the effective use of PACT. The results of the uptake study, presented in Figure 10, demonstrate a clear difference in uptake efficiency between the compounds, with the conjugates exhibiting significantly enhanced cellular accumulation compared with that of free RB.

For the quantitative evaluation of photosensitizer delivery to the cells, cellular uptake measurements were conducted as follows. The uptake of each lectin conjugate was determined at a concentration of 2 µM and calculated relative to that of RB. The uptake of the PSA-RB/LABA-RB mixture was measured using a total photosensitizer concentration of 2 µM, which was composed of 1 µM PSA-RB and 1 µM LABA-RB.

The theoretical additive uptake was calculated as half of the total of the measured uptake values for each of the conjugates using Equation (1):

Theoretical uptake = ½ (uptake_LABA_ + uptake_PSA_) (1)

The theoretical uptake served as the standard for evaluating synergistic or competitive binding in the mixture, as the actual photosensitizer uptake is inherently concentration dependent. This theoretical value represents the expected uptake if the conjugates are bound independently and without interference. This parameter was compared with the experimentally measured uptake of the mixture. This quantitative comparison was essential for determining whether the observed PACT efficacy of the mixture was due to true synergistic binding or additive effects.

In all the cases, the uptake of free RB was significantly lower than that of each conjugate or of the conjugate mixture (Figure 10). For most of the strains tested (excluding *S. aureus*), the measured absorption of the mixture significantly exceeded the theoretical additive value. These findings convincingly demonstrated that the presence of both conjugates promoted more efficient adsorption of both components.

The *S. aureus* results suggested an additive binding mechanism despite the high PACT efficacy. The experimental uptake of the mixture was approximately equal to the theoretical additive uptake. This crucial finding indicates that the LABA and PSA conjugates neither compete for the same binding nor synergize in the physical binding process; rather, each conjugate binds independently. Consequently, the observed significant improvement in PACT efficacy, which was the highest among all pathogens tested, was not driven by an increase in the total quantity of photosensitizers delivered. We propose that the synergistic effect of PACT is related to the complementary and more uniform distribution of the two independent conjugates across the cell surface, which maximizes the area of impact of ROS on target membrane structures.

The data on *P. aeruginosa* uptake revealed that positive synergism was clearly observed in the cellular uptake, since the experimental uptake of the mixture was higher than the theoretical uptake. This finding confirms that the dual-targeting strategy successfully enhances the delivery and accumulation of RB on the cell surface. However, this enhanced delivery did not correlate with an increase in PACT efficacy compared with that of the PSA-RB conjugate when applied alone. We assume that the absence of correlation indicates saturation of the targeted site. Consequently, in this specific system, the concentration of the PSA-RB single conjugate already represents the optimal or excessive dose required to achieve maximal cytotoxicity.

In the case of *S. paratyphi* B, *E. coli*, and both MRSA strains, the strategy of applying both conjugates achieved synergistic enhancements in both uptake and PACT efficacy. For these strains, the measured uptake of the mixture was higher than the expected additive sum. The improved photosensitization can be directly attributed to the increased delivery of the photosensitizer achieved through the synergistic dual-targeting mechanism. This robust correlation establishes the dual-targeting strategy as the most effective route for low-dose PACT against these key pathogens.

This pattern of enhanced uptake and improved PACT performance aligns with previous reports, which demonstrate that the targeted delivery of photosensitizers, whether via lectins or other carbohydrate-binding moieties, significantly increases bacterial accumulation and photoinactivation efficiency compared with free photosensitizers [5,9,30,31].

Despite the antibacterial efficacy of lectin–RB conjugates, their therapeutic application is limited. Lectins are inherently immunogenic proteins, and their systemic administration triggers immune responses in humans and animals [32,33,34,35], which restricts their direct intravenous use as drug carriers. Consequently, the present strategy is not intended for systemic antimicrobial therapy. Instead, lectin-based targeting is particularly well suited for localized or non-systemic applications, such as topical antimicrobial photodynamic therapy (e.g., skin, wound, oral, or dental infections), surface disinfection, or antimicrobial coatings. Within these defined boundaries, lectin-guided photosensitizer delivery represents a powerful and versatile platform for selective antimicrobial targeting without contributing to antibiotic resistance.

## 3. Materials and Methods

### 3.1. Materials

*Pisum sativum agglutinin* (PSA), extracted from *Pisum sativum* seeds, and *Laburnum anagyroides agglutinin* (LABA), derived from *Laburnum anagyroides* bark, were purchased from Lectinotest (Lviv, Ukraine). Rose Bengal (RB) was purchased from Alfa Aesar (Heysham, UK). N-ethyl-N′-(3-dimethylaminopropyl) carbodiimide (EDC) was supplied by Glentham Life Sciences Ltd. (Corsham, UK), and N-hydroxysulfosuccinimide sodium salt (sulfo-NHS) was obtained from Sigma–Aldrich (St. Louis, MO, USA). All chemicals were of analytical grade and were used without further purification. This material list is consistent with that used in our previous study [9].

### 3.2. Bacterial Growth

*Staphylococcus aureus* (ATCC 25923), *Escherichia coli* (ATCC 25922), *Pseudomonas aeruginosa* (ATCC 25668), and *Salmonella paratyphi B* (ATCC 8759) cultures were obtained from ATCC (Manassas, VA, USA). Clinical strains MRSA 4524665 and MRSA 4529137 were kindly provided by Dr. Haim Ben-Zvi and Dr. Esti Michael (Rabin Medical Center, Petach Tikva, Israel). The bacteria were cultured as described previously [9]. Strains were initially grown on brain heart agar (BHA, Acumedia, Lansing, MI, USA) plates for 24 h at 37 °C. Colonies were then transferred to Brain Heart Infusion Broth (BHI, Acumedia) and incubated at 37 °C with shaking at 170 rpm until an optical density of OD_660_ ≈ 0.2 was reached. The bacterial suspensions were subsequently diluted to OD_660_ = 0.10 ± 0.02, corresponding to approximately 10^8^ CFU/mL, and further diluted to 10^4^ CFU/mL for use in all experiments.

### 3.3. Agglutination Study

Agglutination activity was evaluated according to the procedure described by Atrash et al. [9]. Bacterial cultures of *S. aureus*, *E. coli*, *P. aeruginosa*, and *S. paratyphi* B were grown as described in Section 3.2 until they reached an OD_660_ ≈ 0.2. PSA and LABA were dissolved separately in 0.1 M phosphate-buffered saline (PBS, pH 7.4) to a final concentration of 1 µM. For each test, 50 μL of bacterial suspension was mixed with 50 μL of PSA or LABA solution on a clean glass slide. The mixtures were gently stirred using a sterile inoculation loop, and agglutination was assessed microscopically (Olympus CKX53, Tokyo, Japan) after 2 min. Untreated bacterial suspensions served as controls. The appearance of visible clumping indicated positive agglutination, whereas a uniform suspension was interpreted as negative.

### 3.4. Synthesis and Characterization of PSA-RB and LABA-RB Conjugates

The conjugation of Rose Bengal (RB) to PSA or LABA was carried out according to a protocol originally described by Cantelli et al. [10] and adapted in our previous work [9]. RB disodium salt was first dissolved in DMSO to a final concentration of 10 mM. To activate the dye, EDC (27.5 mM) and sulfo-NHS (15 mM) were added under constant stirring, and the mixture was allowed to react for 5 h at room temperature. The activated RB solution was then added to PSA or LABA (0.05 mM), which had been dissolved in 0.1 M sodium carbonate buffer (pH 9.0) beforehand, and the mixture was incubated overnight under gentle stirring. To separate the unbound dye from the conjugated protein, the reaction mixture was purified via gel filtration using Sephadex G-50 and eluted with the same buffer. Conjugates were collected, protected from light, and stored at 4 °C until use.

### 3.5. Photodynamic Antibacterial Activity Assay

The antibacterial activity of free RB, PSA-RB, LABA-RB, and the mixture of conjugates, applied at a 1:1 mass ratio, was assessed as described previously [9]. The concentrations of the conjugates are presented relative to the concentration of RB. Bacterial suspensions of *S. aureus*, MRSA, *E. coli*, *P. aeruginosa*, and *S. paratyphi B* were adjusted to 10^4^ CFU/mL and incubated with different concentrations of free RB or RB/lectin conjugates (1 mL per well) in 48-well flat-bottom plates. Samples were pre-incubated in the dark at 37 °C with shaking (100 rpm) for 15 min. Illumination was performed using an 18 W LED lamp (OSRAM L18W/765, 400–800 nm, 26 mW/cm^2^), positioned 8 cm above the samples. Light doses of 3.1, 7.8, and 23.4 J/cm^2^ were used for *S. aureus* and MRSA, and 46.8, 93.6, and 140.4 J/cm^2^ were used for Gram-negative cells. Dark controls were run in parallel. At selected time points, 0.1 mL aliquots were plated on BHA and incubated overnight at 37 ± 1 °C. Colony-forming units (CFUs) were enumerated using an automated Scan 500 colony counter (Interscience, Saint-Nom-la-Bretèche, France).

### 3.6. Photostability Tests

The photostability was measured in 0.1 M PBS, pH 7.4, in 1 cm quartz cuvettes with Teflon stoppers during irradiation with an LED lamp. Absorption spectra were recorded before light treatment and during irradiation using a Thermo Scientific Evolution 201 spectrophotometer (Thermo Fisher Scientific Inc., Madison, WI, USA). The initial optical densities of the RB and conjugates at the absorption maximum were ∼0.2. The ratios (A_t_/A_0_) of the measured absorbencies at the long-wavelength maximum before (A_0_) and after irradiation (A_t_) were taken to plot decay curves. The photostability experiment for each sample was performed 3 times, and the average decay curve was obtained.

### 3.7. ROS Generation

The quantum yields of singlet oxygen generation (Φ_Δ_) of conjugates in 0.1 M PBS, pH 7.4, with ABMDMA as the scavenger were measured. RB was used as the reference dye, with a yield of 0.76 [36]. The solutions were illuminated in standard 1 cm quartz cuvettes with Teflon stoppers using an LED lamp, and the absorption spectra were recorded over time. The absorbance plot of ABMDMA at 401 nm vs. time was drawn and fitted by a first-order reaction rate function. The values of Φ_Δ_ were calculated via the following formula [36,37].

Φ_Δ_(lectin–RB) = Φ_Δ_(RB) × *k*_lectin–RB_/*k*_RB_

where Φ_Δ_(RB) and Φ_Δ_(lectin–RB) are the quantum yields of the singlet oxygen generation of RB and conjugates, respectively, and *k* are rate constants from the corresponding fitting curves. The experiment for each sample was performed 3 times, and the average was obtained.

### 3.8. Bacterial Uptake of RB and RB–Lectin Conjugates

Suspension of bacteria in saline (10^7^ cells/mL, 3 mL) was incubated with 2 μM RB or lectin–RB conjugates for 30 min at 37 °C, after which the absorbance was measured. Then, the cells were separated by centrifugation (6000 rpm for 10 min), and the supernatant was collected and re-measured. The dye uptake was calculated as follows:

Uptake (%) = (1 − A_supernatant_/A_initial_) × 100%

where A_supernatant_ is the absorbance of the supernatant, and A_initial_ is the absorbance of the bacterial suspension before centrifugation.

### 3.9. Statistical Analysis

The results obtained from at least three independent experiments, which were carried out in duplicate, were analyzed using single-factor ANOVA. The difference between the results was considered significant when the *p* value was less than 0.05. Quantitative results are presented as the means ± standard errors.

## 4. Conclusions

This study demonstrated that the conjugation of Rose Bengal (RB) with specific plant-derived lectins, *Pisum sativum* agglutinin (PSA) and *Laburnum anagyroides* agglutinin (LABA), significantly enhances the photodynamic antibacterial efficacy of RB. Compared with the free photosensitizer, the synthesized PSA-RB and LABA-RB conjugates retain high singlet oxygen generation capacity and exhibit improved photostability.

Photodynamic inactivation assays revealed that PSA-RB is broadly effective against both Gram-positive and Gram-negative bacteria, including multidrug-resistant strains of MRSA. LABA-RB, while less potent alone, contributes to enhanced efficacy when used in combination with PSA-RB. The binary conjugate mixture exhibited a strong synergistic effect, resulting in complete bacterial eradication at significantly lower concentrations and light doses than either the agent alone or free RB.

These results support the hypothesis that dual-targeted lectin conjugation enables more efficient accumulation of RB at the bacterial surface, thereby amplifying the photodynamic effect. This approach offers a promising alternative to conventional antibiotics, especially in the treatment of infections caused by resistant pathogens. The data obtained confirm the potential of the multiple-target binding strategy in antimicrobial photodynamic therapy, opening new prospects for the development of effective agents against antibiotic-resistant bacteria.

## Data Availability

The data supporting the findings of this study are available in the current publication.

## Abbreviations

The following abbreviations are used in this manuscript:

ABMDMA	9,10-anthracenediyl-bis(methylene)dimalonic acid
LABA	*Laburnum anagyroides* agglutinin
MRSA	Methicillin-Resistant *Staphylococcus aureus*
MSSA	Methicillin-Susceptible *Staphylococcus aureus*
PACT	Photodynamic Antibacterial Chemotherapy
PSA	*Pisum sativum* agglutinin
RB	Rose Bengal
